# Intelligent Calibration of Static FEA Computations Based on Terrestrial Laser Scanning Reference

**DOI:** 10.3390/s20226439

**Published:** 2020-11-11

**Authors:** Wei Xu, Xiangyu Bao, Genglin Chen, Ingo Neumann

**Affiliations:** 1 Geodetic Institute, Leibniz Universität Hannover, Nienburger Str. 1, 30167 Hannover, Germany; xiangyubao0430@gmail.com (X.B.); neumann@gih.uni-hannover.de (I.N.); 2 School of Electrical & Power Engineering, China University of Mining & Technology, Xuzhou 221116, China; ccglcumt@126.com

**Keywords:** terrestrial laser scanning, finite element analysis, deep learning, long short-term memory, calibration, sequence

## Abstract

The demand for efficient and accurate finite element analysis (FEA) is becoming more prevalent with the increase in advanced calibration technologies and sensor-based monitoring methods. The current research explores a deep learning-based methodology to calibrate FEA results. The utilization of monitoring reference results from measurements, e.g., terrestrial laser scanning, can help to capture the actual features in the static loading process. We learn the deviation sequence results between the standard FEA computations with the simplified geometry and refined reference values by the long short-term memory method. The complex changing principles in different deviations are trained and captured effectively in the training process of deep learning. Hence, we generate the FEA sequence results corresponding to next adjacent loading steps. The final FEA computations are calibrated by the threshold control. The calibration reduces the mean square errors of the FEA future sequence results significantly. This strengthens the calibration depth. Consequently, the calibration of FEA computations with deep learning can play a helpful role in the prediction and monitoring problems regarding the future structural behaviors.

## 1. Introduction

The field of artificial intelligence has been developing rapidly in recent years [1]. It has made significant contributions regarding the optimization and prediction of many problems [1,2,3]. The machine learning-based approach has proved to be a suitable method to solve the finite element analysis (FEA) calibration problem [4]. However, its accuracy and calibration depth still need to be improved significantly. Therefore, this paper focuses on the FEA calibration to further improve its efficiency, depth, and accuracy by utilizing the advanced deep learning approach.

### 1.1. Development of Classical FEA Calibration

The calibration of the FEA is a process of tuning and improving the FEA computation, which aims to predict and analyze the future behavior of the object better. Efficient methods regarding FEA calibration have been reported in many cases [5,6,7]. Abrahamsson and Kammer applied the experimental frequency response to calibrate the dynamic FEA model [5]. The damping equalization is carried out to solve problems regarding the mode pairing in experiments and computations. It combines the model reduction to improve the efficiency. The results indicate that the application of the frequency response offers a scientific and reliable calibration deviation metric. Different calibration methods combining dynamic experimentation have been presented, including the Levenberg–Marquardt minimizer [6] and the genetic algorithm [7] to optimize parameters. Additional calibration studies of complex structures were carried out based on the fundamental principles above. By comparing the deviations between the experimental and numerical data, Abrahamsson et al. researched on the front subframe model of the car with complex degrees of freedom [8]. They proposed a cross validation method by combining both Levenberg–Marquardt and Gauss–Newton minimizers. Osmancikli et al. calibrated the computational dynamic characteristics by the experimental characteristics in the ambient vibration test by changing the stiffness coefficients of the connection joints of the initial FEA model [9].

The long-span suspension structure is one of the most advanced techniques in constructing bridges which can be found as important traffic roads built over the long-span river, lake or sea. Wang et al. developed two phase models to solve the calibration of long-span suspension bridge [10]. They simplify the entire progress of the calibration by the later combination with the separated vibration and static measurements. Garo et al. created a method to calibrate the material property in FEA computation by using an inverse method based on the experimental structure performance at different dynamic loadings [11].

The methods above apply a single parameter to calibrate FEA computations. The multivariate-based FEA calibration is more challenging. Formisano et al. proposed the calibration based on the natural frequencies and damping coefficients in the ABAQUS software environment [12]. A reliable calibration process is carried out in the commercial FEA package ABAQUS by obtaining the required inner structural properties [13]. A set of calibration results, including the loading-displacement response, the strain distribution, and the failure mode are presented through the indentation test with complex mixed mode loadings. A good displacement agreement in the results of both the FEA computation and the experiment has been reliably confirmed. Chen et al. used a continuous health monitoring system in the bridge [14]. The output model resulting from the ambient vibration experiment calibrated the FEA model and improved the correlation between the computational and experimental parameters. Erdogan calibrated the discrete and continuous FEA models thorough the combination of vibration and material tests for the purpose of the seismic assessment [15]. The procedure considers the stiffness of contact between adjacent stone units. The use of experimental data provides a sufficiently reliable calibration of the FEA. An inverse technique, called the virtual field method, calibrates the FEA material model by tracking the parameter covariance of large errors and the existence of equivalent parameters [16]. As the number of variate parameters increases, the classical FEA calibrations become increasingly time-consuming. Therefore, new calibration techniques with machine learning are increasingly gaining interest in the FEA calibration community.

### 1.2. FEA Computation and Calibration with Machine Learning

The FEA computations and calibrations now apply the advanced machine learning techniques. The latter is a powerful tool to understand the FEA processes and provide calibration solutions based on the deviation results between the experimental and FEA data. It can solve extraordinarily complex and difficult tasks for many engineering applications. Machine learning permits computers to learn the changing laws and behaviors based on the trained data [17,18,19,20]. With the combination of the FEA, machine learning can learn from the complicated patterns and understand the FEA process. There are lots of integration methods regarding the FEA and machine learning which one can apply in computations of modal characteristics, finite elements, constitutive relationships, material parameters, the FEA geometric deviation, and mechanical behaviors. There is an increasing need for fast and accurate computations and calibrations in the FEA.

Combined with response surface and particle-swarm methods, Marwala implements supervised learning in the form of Bayesian neural networks (NNs) which calibrate the mode and frequency domains in FEA [21]. The Markov chain Monte Carlo technique solves the Bayesian formulation well. The mode shape and frequency domains in the FEA computation are calibrated in NNs according to the model assurance criterion and natural frequencies tested.

Applying machine learning determines the FEA element state in both regular and multiscale structure problems [22]. The novel model regarding the smart finite elements extracts data from the FEA which is fed into the machine learning algorithm later, avoiding the complex task of finding the displacement field and complex iterations in the original computation. Accordingly, this reduces both the computational cost of producing and the computational error significantly.

Nie et al. explored a deep learning strategy to accelerate the computation of constitutive relationships [23]. Convolutional neural networks (CNNs) are beneficial in determining the FEA stress field regarding a two-dimensional cantilevered structure. This novel approach takes the object geometry, loads, and displacement boundary as inputs to output the predicted computational stress results. Oishe and Yagawa described computational mechanics and implicit rules regarding stiffness matrices through deep learning enhancement [24] Deep learning-based strategies indicate the robustness and flexibility. They also introduce more opportunities to apply in more complex FEA problems.

Javadi et al. calibrated the conventional material parameter model in FEA regarding the embankment through applying an artificial NNs-based methodology [25]. The relationship of the Mohr–Coulomb material subjected to loadings is outputted and calibrated. Similarly, Javadi et al. proposed an artificial intelligence regarding the evolutionary polynomial regression to calibrate complex material parameters in the FEA computation [26]. Hence, the optimization and calibration process of internal materials are also displayed to readers more intuitively.

Advances in the FEA computation have also been preliminarily applied in soft-tissue biomechanics and human organs. However, discussing the irregular and complex structures is too computationally burdensome for the application in real-time applications. The combination of the FEA with machine learning can offer an efficient solution to solve this problem. Reducing the computational cost and complexity of FEA is one of the main topics in the medical application by utilizing different techniques, for example, NNs-based strategy [27] and regression models-based prediction [28]. A deep auto encoder approximates the behavior of a non-linear and muscle actuated beam structure [29]. The deformation space in the compact form regarding high degree soft tissue is calibrated by Artisynth’s quasi-static incremental solver through the use of deep learning models. The application of the auto encoder deep learning approach indicates a lower reconstruction error, which is compared with the original FEA model. The similar idea can also be applied to estimate, calibrate, and recover the zero-pressure geometry of the patient’s thoracic aorta is developed on the basis of the FEA method [30]. The application of different machine learning methods can simplify, predict, and calibrate the complex FEA computations, thus, solving medical problems.

Liang et al. integrated the principal component analysis, sparse coding, nonlinear regression, and bidirectional neural networks to study the shape characteristics and Von Mises stress behavior of FEA computation [31]. The inputs are the material parameters based on experiments and statistical geometric shape. The calibration and optimization from FEA results enable the output stress analysis of human organs into fast speed, reliable, and real-time analysis. Li et al. recently designed an encoder–decoder-based CNN-FEA model [32]. Inputting geometry features and FEA boundary conditions result in the prediction of time-dependent concentration distributions. The mean relative error is very low, which indicates that the test accuracy is very high in the novel model.

However, the methods above ignore the depth of the FEA calibration. They mainly focus on one-step based calibrations. All these effective studies indicate that machine learning can greatly capture the complex behaviors of FEA computation and provide calibration possibilities to improve the FEA. Automatic and smart algorithms train and simulate the complicated behaviors and computational processes in FEA. Consequently, intelligent algorithms can make predictions and decisions about the future contour without being explicitly programed in the mathematical FEA calculation. Combining the FEA with deep learning, predictions regarding the future behavior of images or next frames and their calibrations are possible directions in this field, in which one seldom finds future image predictions based on the known FEA pattern data. The depth of the prediction can be extended through the application of deep learning. Future image prediction is also known as the next-frame prediction in the video generation, which is discussed in the next section mainly.

### 1.3. Deep Learning

Deep learning is a subset of machine learning which commits to research on computations and analysis with intelligence. Practical applications regarding deep learning in the context of patterns or images processing has increased tremendously increase in recent years [33,34,35,36,37]. The generic deep learning contains various methods of learning and predicting future images, including recurrent neural networks (RNNs), CNNs, and others [38,39,40]. They help one to learn and understand the dynamic mechanisms process and characteristics of the object under consideration and feedback future predictions from learning. The image sequence prediction is a challenging task which involves understanding continuous motion images at different levels. This is similar to the way in which humans can forecast anticipated changes through the cooperation of brain neurons and sensory perception. Consequently, the future sequence image prediction is sufficiently helpful to further learn the FEA behavior patterns, for example, the displacement, stress, and strain patterns. The prediction theory illustrates the interaction of feed-forward and backward information flow [41]. Many advanced vision applications benefit a lot from the knowledge of future image prediction. The application of future image predictions has been widely utilized in automatic car-guidance [42], prediction of the moving target’s position [43], robotic motion [44], weather forecasting [45,46], and video-frame prediction [47,48].

Srivastava et al. utilized long short-term memory (LSTM) networks with multi-layers to learn video image sequences, which predicts future sequence by decoding multiple LSTMs [49]. The introductions regarding LSTM details and its theories are in Section 2.3 and Section 3.2. LSTM is a widely applicable kind of RNN which contains feedback connections for both single data points and entire data sequences in deep learning [50]. The optimization task regarding accurate future image prediction has been a highlighted problem in artificial intelligence in recent several years [51,52,53,54,55,56,57,58,59,60,61,62,63,64,65,66,67]. Kalchbrenner et al. have developed a video pixel network to predict the joint distribution of future image in pixel videos [60]. It encodes a four-dimensional dependency chain to reflect different space, time, and color structures. Only minor deviations from the ground truth are indicated with the application of this prediction model. Xue et al. proposed a cross convolutional network to synthesize future images in a probabilistic manner, based on auto-encoders of future maps and convolutional kernels, respectively, with the single input image and unknown motions [52]. Additionally, subsequent layers model [66], generative adversarial networks [56], CNNs [55], convolutional LSTM [68], and cubic LSTM [58] play significant roles in the prediction of future images.

Summarily, the input of the prediction task is the image data regarding the current and previous status. The aim is to output and predict the possible future images. The kernel insight of all these applications is to learn the variant rules of images and predict how the research object will change from visual images accurately over time. There are similar features and powerful reference values between the future image prediction topic and the computational patterns prediction in the continuous static FEA behavior computations. What they significantly describe and contain in common are the variant patterns over continuously different loadings. One of the final aims of this research is to provide fast calibrated image sequence results of FEA behavior computations using the benefit of the prediction technology regarding future images. Deep FEA calibration is another aim. Consequently, this research selects the LSTM method to predict and calibrate sequence results in the static FEA.

## 2. Motivation and Methodology

### 2.1. Motivation

There are many analysis and optimization tasks in mechanical and structural engineering and fluid dynamics which use FEA computations as their basis. With the development of relevant engineering applications regarding the FEA calculation, the scale of calculations is becoming greater and increasing rapidly. A generic approach to carry out FEA computations is to apply simplified models in the computation of large composite structures, which provides us sufficient efficiency to obtain the characteristics of the researched object quickly [69]. A significant problem is also indicated that the accuracy of FEA computations based on the simplified model is insufficient. As is discussed in [69], the parametric method, for example, the B-spline method, which is based on the laser scanning data is proposed to calibrate the simplified FEA computation. This kind of method can indeed optimize the FEA computation results for the accurate parametric representation of the scanning sensor data regarding the object structure. Expensive time costs are indicated through complex computations and complex geometries. Consequently, the efficiency is greatly affected utilizing this method while the accuracy is improved a lot. The FEA computation time varies from hours to weeks in some engineering composite structures with large scales, which makes the FEA insufficiently efficient. The problem is that there need to be a balanced approach to integrate the accurate calibration reference with the efficient standard FEA.

Deep learning is considered to be a novel approach to balance the efficiency, step size, and accuracy of the FEA calibration. The idea of deep learning models is to learn from the accurate and more complex FEA computation or other accurate measurements. The training process itself is time consuming for large amount of training feeding in the deep algorithm. However, with the application of deep learning, the computation of the implicit rules, material properties, complex boundaries conditions, and physics equations can be learned effectively once the training is accomplished, as is described in Section 1.3. It is still a rare combination to apply deep learning to the FEA computation. Therefore, it is in a starting stage in this scientific combination aspect.

The FEA post-processing with high efficiency and quality is a significant advantage concerned by researchers. Post-processing results provide us with an intuitive visual representation of the mechanical characteristics, for example, the displacement, stresses, and strain contour. Our methodology is inspired by the visual representation of the mechanical data in the FEA post-processing and the strong data-processing power of deep learning. Therefore, we expect that both efficiency and accuracy of the FEA calibration can be maintained with the new deep learning-based method.

### 2.2. Framework

Section 1.1 and Section 1.2 indicate that the mature frameworks of FEA calibrations regarding constitutive relationships, material parameters, and mechanical characteristics are generally based on the response between inputs and outputs. Meanwhile, multi-sensors data or experiment-based data is the fundamental of the calibration. Different optimization techniques can be applied according to various calibration goals. Therefore, the first task in the current research is to find out a scientific solution to address the gap which is indicated in the motivation section. By clarifying the calibration objective, we can apply deep learning to establish a suitable algorithm between the real and FEA data for effective calibrations. The FEA post-processing data regarding the mechanical characteristics, including the stress, strain, and displacement corresponding to various loadings, is one of the calibration features concerned in the FEA application process.

**Figure 1:**
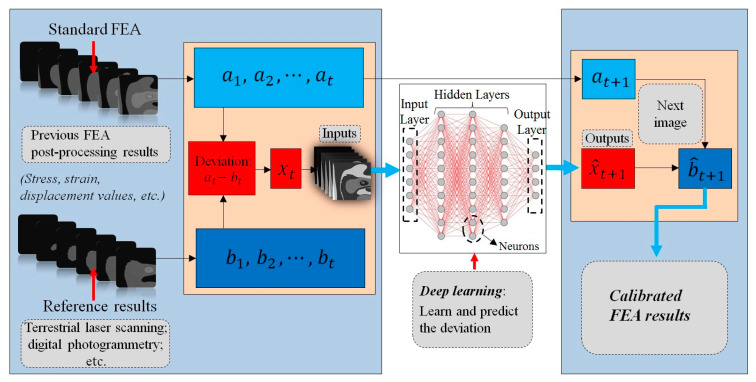
Framework of the proposed methodology.

The data-processing of deep learning and visual representation of mechanical data in the FEA combines the framework of the current methodology, as is shown in Figure 1. The entire framework is composed of three parts, which are the preparation of database, deep learning of the model, and the final generation of the calibrated FEA results. The calibration database is derived from two groups of images. One is from the output of the standard FEA to be calibrated, which is represented as $a_{t}$. Here, the standard FEA means the normal FEA computation with the simplified geometry or rough parameter settings. Another is from the output of reference value, which is represented as $b_{t}$. The given inputs in the deep learning process are necessary to finally predict the desired output, as is described in Section 1.3. Using a similar solving process, the input dataset for the training task in this methodology is the deviation results between the standard FEA and calibration reference values, which can be based on the displacement, stress, and strain in different cases. The network structure displays and computes the deep learning hierarchically. The architecture schematic of NNs is drawn by the online platform AlexLenail NN-SVG [70]. Multiple layers are the core of deep learning to execute the computation. A layer is defined logically as a separated group of neurons in deep learning. Neurons in the first layer, which is also called the input layer, receive the information and features from the input data steam. Input features are extracted from the deviation calculation with time series. All input data are converted to pixel values in a set of arrays. The last layer in the model is the output layer which executes the output of the required data. There are multiple hidden layers between the input and output layers. The ratio regarding the training and validation datasets depends on the deep learning method. Details about the deep learning datasets in this research are explained in Section 3.3.2. Each layer in the deep learning model plays different roles, for example, abstracting the pixels, encoding, and decoding. Deviation-based results are learned and calibrated through the training process. The deep learning model outputs the predicted deviation results. The calibrated FEA results are calculated by integrating the next image generated from FEA computation and the predicted deviation result finally. The NNs input, output, and final calibrated FEA values in this research are related to the equivalent stress values, which are represented as gray intensity images in all the subsequent sections. Requirements regarding the calibration reference method should be easy-to-access and graphical display. The given loadings, boundary conditions, material parameters, and the simplified geometric model provide the standard FEA computations. The calibration reference monitoring should be processed under the same conditions to ensure the comparability of the final results.

The process of the original FEA and reference values is shown in Section 3.3.1. The calibration process is composed of two chains overall. The standard FEA computation is remained as the first chain for its fast and efficient computation with the simplified model. The framework focuses on the deviation calibration from the reference results. Hence, the second chain is to predict an accurate deviation. The calculation of the deviations is presented in Section 3.3.2. The implementation of deep learning captures the variation characteristics regarding the deviation values. The integration of the standard FEA result and the predicted deviation value determines the final calibration values. The application of this methodology solves the problems in the motivation section. The training process of deep learning is complex and time-consuming. However, the deep learning model when finally trained can perform an accurate prediction quickly and be utilized efficiently. This framework realizes both the efficiency and accuracy of calibrated FEA results.

There are some choices for the reference dataset. Terrestrial laser scanning (TLS) and digital photogrammetry are advanced measurements and monitoring tools that can provide reference patterns to detect the surface information accurately [71,72]. With the application of TLS, the surface information regarding the object can be measured rapidly as the point cloud data with high precision [73,74]. The precision of TLS can reach the level of ±1 mm and its resolution at the industrial environment is 10,000 Pixel/360° and even higher [73]. Digital photogrammetry is also an advanced technique to acquire three-dimensional geometric information from stereoscopic image overlap [75,76]. Both measurement methods have extensive engineering applications in a variety of fields. This manuscript uses a TLS-based monitoring method because its precision is very high and acquiring large amounts of point clouds is achieved quickly. The reference in this research is the parametric model-based FEA described in [69]. It is a FEA computation on the basis of the TLS data which refers to the TLS-based reference in the next sections. The point cloud from TLS is approximated by B-spline surfaces. The advantage of parametric model-based FEA is that it provides a high volume of database of various computational patterns from a large number of computations. The accuracy of the parametric model, which is based on the B-spline surface approximation, can be highly improved by the FEA computation [69].

Various techniques are utilized for the training process regarding the addressing sequence data, as is described in Section 1.3. LSTM is one of the most highlighted techniques in fixing long sequence training and is well known in predicting continuous future steps in movie frames [68] and weather forecasting [45]. Moreover, this technique ensures and improves the prediction accuracy of the model through the development in recent years. Therefore, it is applied as the main technique to learn, predict, and calibrate the sequence data regarding mechanical characteristics in the FEA computation. Section 2.3 shows details of the general LSTM implementation.

### 2.3. LSTM Implementation

**Figure 2:**
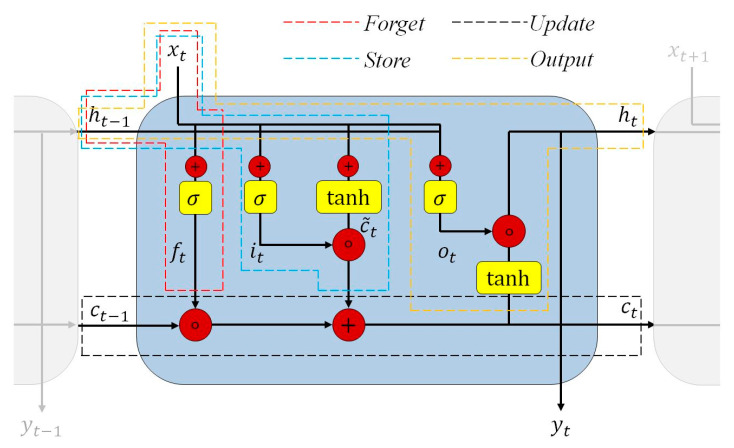
The repeating module of a standard long short-term memory (LSTM) model.

The LSTM network is a special structure in RNN, which was firstly proposed by [50] in 1997. It is capable of learning various practical mechanisms regarding spatiotemporal information. The calibration problem of FEA results are described as a spatiotemporal problem if the FEA results are divided into tiled non-overlapping slice-images containing pixel measurement results. The FEA calibration is transformed into the spatiotemporal prediction. Therefore, LSTM is selected as the implemented deep learning approach to learn, calibrate, and predict the future images of FEA. LSTM contains the form of repeating module chain with various computational blocks, as is shown in Figure 2. It is different with the standard RNN, for standard RNN contains a simple neural net layer to perform the computation. LSTM is more complex in its repeating unit with different layers. These layers are computational blocks which interact to selectively control the information flow within the cell. Therefore, LSTM cells are capable to track related information throughout time steps.

One of the most significant components of LSTM is the gate. Related information can be removed or added through gates in NNs optionally. LSTM processes information through four steps. Each step is marked with various dashed blocks in red, blue, black, and orange colors in Figure 2.

(1) $$f_{t}=\sigma \left(W_{xf}x_{t}+W_{hf}h_{t-1}+b_{f}\right)$$

(2) $$\sigma \left(x\right)=\frac{1}{1+exp\left(-x\right)}$$

The first step is to decide what information is going to be thrown away from the cell state. This task regarding forgetting the irrelevant history is finished by a sigmoid layer named as the forget gate layer, which is marked as a red dashed block. Equation (1) describes the computational relationship. It is a function of the prior internal state$\;h_{t-1}$ and the current input$\;x_{t}$, which outputs a number in the range of [0, 1] by a sigmoid function. Equation (2) represents the calculation of sigmoid function σ. The number of 0 means to discard it completely, while the number of 1 means keep it completely. Consequently, the forget gate function can decide whether the information is important. The parameters regarding W and b in all following contexts and equations stand for the weight and bias for the respective gate neurons, which are learnable parameters.

(3) $$i_{t}=\sigma \left(W_{xi}x_{t}+W_{hi}h_{t-1}+b_{i}\right)$$

(4) $${\tilde{c}}_{t}=tanh\left(W_{xc}x_{t}+W_{hc}h_{t-1}+b_{c}\right)$$

(5) $$tanh\left(x\right)=\frac{exp\left(x\right)-exp\left(-x\right)}{exp\left(x\right)+exp\left(-x\right)}$$

The next step is that LSTM can decide what part of the new information is relevant. This will be utilized to store the information into its cell state. This is the store layer with two sub chains which are marked with a blue dashed block in Figure 2. One chain$\;i_{t}$ with a functional relationship in Equation (3) is computed with a sigmoid function to update the prior internal state$\;h_{t-1}$ and the current input$\;x_{t}$. The computation of this chain squishes the value between 0 and 1. Anther chain is computed with a tanh function in the range of −1 to 1 to pass $\;h_{t-1}$ and$\;x_{t}$, as is shown in Equation (4). Equation (5) describes the computation of the tanh function. The aim of this tanh chain is to create a new candidate based on the regulation of the parameters in the neural network. Finally, the sigmoid chain decides which information from the tanh chain is relevant and important to keep and store.

(6) $$c_{t}=f_{t}\circ c_{t-1}+i_{t}\circ {\tilde{c}}_{t}$$

The third step is to calculate and update the cell state, illustrated in the black dashed block in Figure 2. It is called the update layer and performs both actual updating and forgetting. The Hadamard product is represented as ∘ in this research. The cell state$\;c_{t}$ is an important conveyer belt which extends along the entire chain. The state in the network is a sort of simplifying. There are a group of neurons which are firing the numbers squished by the sigmoid or tanh functions. The weight and biases constantly modify and compute these numbers. Hence, those numbers hold a state. Equation (6), which is separated into two parts, computes the updating of the cell state. Firstly, the latest previous cell state$\;c_{t-1}$ is calculated with the output$\;f_{t}$ from the forget layer. The previous state is used selectively and can be forgotten in this process when the forget layer outputs a value near 0. Secondly, the calculation of results from two sub chains of the store layer is processed. The new cell state is computed by the sum of both parts. In the current paper, the previous FEA deviation results are remembered or propagated together through$\;x_{t}$,$\;h_{t}$ and$\;c_{t}$.

Finally, the calculation and return of the output layer takes place, illustrated in the orange dashed block in Figure 2. It controls what information encoded in the cell state is processed to the network as the input in the next time step. This layer outputs the next hidden state$\;h_{t}$ which is also used for the prediction$\;y_{t}$. This is the predicted FEA deviation result in the next step. The function$\;{ο}_{t}$ feeds the previous hidden state$\;h_{t-1}$ and the current input$\;x_{t}$ into the sigmoid function, described in Equation (7). The updated cell state$\;c_{t}$ is also passed to a tanh function in the final calculation. Therefore, Equation (8) then shows the generation of the output.

(7) $${ο}_{t}=\sigma \left(W_{xο}x_{t}+W_{hο}h_{t-1}+b_{ο}\right)$$

(8) $$h_{t}=y_{t}={ο}_{t}\circ tanh\left(c_{t}\right)$$

Summarily, the LSTM maintains a conveyer belt, called the cell state, throughout the entire network. Gate layers control the information flow of the network, which contains the forget layer, store layer, update layer, and output layer. The most important advantage of LSTM during processing sequences is that the network can regulate and choose what information is relevant to keep or irrelevant to discard efficiently. Hence, LSTM is a highly efficient approach and superior choice to deal with the sequential information and problems throughout time steps. In this research, the inputs are the equivalent stress deviation results computed from standard FEA results and TLS-based reference values. The final outputs in LSTM model of this research are corresponding to the predicted deviations in next few steps of the FEA computations.

## 3. Model

### 3.1. Mathematical Description

The kernel of the intelligent FEA calibration is the prediction of the future result of the FEA computation. The future result prediction is the problem of estimating future FEA images and evaluating the accuracy with known past FEA results. Therefore, it is significant to find an approach to generate next future images. Different input loadings over time determine a group of deformation or stress images in FEA computational results. Here, one considers the static structural FEA computations with an invariant load interval, for example, F,  2F, ⋯, tF. The input object of deep learning is the deviation dataset.

(9) $$a\in \left\{a_{1},\;a_{2},\cdots ,\;a_{t}\right\}$$

(10) $$b\in \left\{b_{1},\;b_{2},\cdots ,\;b_{t}\right\}$$

(11) $$x=a-b\in \left\{x_{1},\;x_{2},\cdots ,\;x_{t}\right\}$$

(12) $$p=p\left(x_{t+1}|x_{t},\;x_{t-1},\cdots ,\;x_{1}\right)$$

Two groups of FEA results, which contain both the standard FEA computation$\;a_{t}$ in Equation (9) and the parametric model-based FEA computation$\;b_{t}$ in Equation (10), determine the deviation. As is discussed in Section 2.2, this methodology can compute and process many features including the stress, strain, and displacement. The equivalent stress values are mainly researched in the current research. The deviations of the values and contour lines regarding the equivalent stress in different computational models are obvious. Both groups of data$\;a_{t}$ and$\;b_{t}$ represent the equivalent stress values from standard FEA computations with the simplified geometry and reference results in Figure 1. Equation (11) describes the calculation of the deviation. The next image of the future FEA deviation result between two computations is$\;x_{t+1}$. The problem can be described by Equation (12), which means the unknown probability distribution p of the next image depends on all of the past FEA images.

(13) $${\hat{x}}_{t+1}=arg\;\underset{x_{t+1}}{\max}\;p\left(x_{t+1}|x_{t-1},\;x_{t-2},\cdots ,\;x_{1}\right)$$

The FEA computation generates the past images. The main aim is to predict the future image$\;x_{t+1}$ with an efficient approach. Therefore, the current task in this research is clear. It is necessary to find the functional relationship of generating future predictions$\;{\hat{x}}_{t+1}$. Equation (13) formulates the relationship to find the possible prediction by maximizing the probability function in Equation (12). However, the probability function in this example is difficult to solve with a precise mathematical solution. A reasonable solution regarding this problem is to search for the minimum of the deviation between the estimated and ground truth values.

The neural network method implements the estimation problem in this research. Stacking consecutive past images sequentially predicts the future image$\;x_{t+1}$. Following this idea, Equation (14) presents the generation in the neural network of the estimated image.

(14) $${\hat{x}}_{t+1}=f\left(x_{t},g\left(x_{t},\;x_{t-1},\cdots ,\;x_{1}\right)\right)$$

Here, the functional generator of the intelligent estimation is f. The current computed FEA image is$\;x_{t}$. The current latent cell of the neural network is g which is associated with the memory of the past computed states$\;x_{t-1},\;x_{t-2},\cdots ,\;x_{1}$. The calculation of g makes the neural network structure to be active in motion. The learning process in this neural network is to optimize the functional generator f. Equation (15) shows that it is necessary to minimize the deviation$\;\Delta x_{t+1}$ between the estimated value and the monitored real data to bring the generated value$\;{\hat{x}}_{t+1}$ close to the real one$\;x_{t+1}$.

(15) $$min\left(\Delta x_{t+1}\right)=\underset{\Delta }{\min}\left|{\hat{x}}_{t+1}-x_{t+1}\right|$$

The deviation between the estimated and real data is represented as the loss function L in Equation (16) to be minimized in the NNs. The loss function should be determined so that the probability distribution results converge to p as the loss function is minimized. Mean square error (MSE) is chosen to be the loss function in this paper as it is easier, more effective, and faster to be implemented in this research. Hence, the model calculates and minimizes the MSE value between the original FEA result and calibrated result. Equation (17) shows the calculation of the final calibrated FEA result.

(16) $${\hat{x}}_{t+1}=arg\;\underset{{\hat{x}}_{t+1}}{min}\;L\left({\hat{x}}_{t+1},x_{t+1}\right)$$

(17) $${\hat{b}}_{t+1}=a_{t+1}+{\hat{x}}_{t+1}$$

### 3.2. Neural Network Model

The deviations regarding the equivalent stress results are monitored over different loads. A pixel representation records each monitoring image with the RGB channel. Each contains M×N grids. From the point of spatial view, the entire g FEA computation can be described as three-dimensional tensors. The tensor$\;X\in {\mathbb{R}}^{P\times M\times N}$ describes each monitored feature. P is the number of the feeding epochs. ℝ indicates the tensor domain of the monitored features which is recorded periodically. Consequently, Equation (18), formulated from Equation (13), transforms the prediction and calibration problem in FEA computation to a spatiotemporal forecasting problem. It indicates that the last length-J FEA computational results generate future length-K predictions with the highest probability.

(18) $${\hat{X}}_{t+1},{\hat{X}}_{t+2},\;\cdots ,{\hat{X}}_{t+K}=\underset{X_{t+1},X_{t+2},\;\cdots ,X_{t+K}}{arg\;max}p\left(X_{t+1},X_{t+2},\;\cdots ,X_{t+K}|X_{t},X_{t-1},\cdots ,X_{t-J+1}\right)\;$$

A solution to the spatiotemporal weather forecasting problem using convolutional LSTM is proposed in [45]. With the comparison of the conventional LSTM [77] extends the convolutional structures regarding both input-to-state and state-to-state. The innovation of convolutional LSTM is that the inputs, outputs, and states are three-dimensional tensors with timestamp, row, and column. The timestamp indicates the spatiotemporal extension of the image-variate information. Equations (19) to (23) show the kernel equations of convolutional LSTM described in [45].

(19) $${ℱ}_{t}=\sigma \left(W_{Xℱ}*X_{t}+W_{ℋℱ}*{ℋ}_{t-1}+W_{Cℱ}\circ C_{t-1}+b_{ℱ}\right)$$

(20) $${ℐ}_{t}=\sigma \left(W_{Xℐ}*X_{t}+W_{ℋℐ}*{ℋ}_{t-1}+W_{Cℐ}\circ C_{t-1}+b_{ℐ}\right)$$

(21) $$C_{t}={ℱ}_{t}\circ C_{t-1}+{ℐ}_{t}\circ tanh\left(W_{XC}*X_{t}+W_{ℋC}*{ℋ}_{t-1}+b_{C}\right)$$

(22) $$O_{t}=\sigma \left(W_{XO}*X_{t}+W_{ℋO}*{ℋ}_{t-1}+W_{CO}\circ C_{t}+b_{ο}\right)$$

(23) $${ℋ}_{t}=O_{t}\circ tanh\left(C_{t}\right)$$

In these, $X_{1},X_{2},\;\cdots ,X_{t}$ are the inputs in the convolutional LSTM, $\;C_{1},C_{2},\;\cdots ,C_{t}$ are the cell outputs, $\;{ℋ}_{1},{ℋ}_{2},\;\cdots ,{ℋ}_{t}$ are the hidden states. The weight is W. The forget gate is$\;{ℱ}_{t}$. The output gate is$\;O_{t}$. All these parameters belong to the three-dimensional tensor of$\;{\mathbb{R}}^{P\times M\times N}$. The input gate in convolutional LSTM is$\;{ℐ}_{t}$. The convolution operator is represented as ∗. The convolution operator in convolutional LSTM replaces the general multiplication operator in conventional LSTM. Details and explanations can be found in [45]. As the input data in LSTM networks is one-dimensional, the spatial image sequence data is impossible to predict with LSTM. Convolutional LSTM can capture underlying spatial image features by applying convolutional operators. Therefore, the convolutional LSTM model reasonably combines the characteristics of convolutional networks regarding the image recognition and LSTM networks regarding ‘memorizing past’. Another dramatically different point between the convolutional and standard LSTM networks is the additional calculation term$\;W_{C}\circ C$ added into forget, store, and output layers. It is called the peephole connection [78]. Our convolutional LSTM implements peephole connections. Traditional LSTM, illustrated in Figure 2, indicates the problem that some information is potentially lost during the computation of those gates for the loss of direct connections from the cell state. Therefore, the calculations of these gate layers can look at the cell state with the peephole connections after computation. These gates with peephole connection can understand unwanted inputs and error signals. The advantage of using the cell state to control different gates is that the gradient is prevented from vanishing too fast because it is already trapped and computed through the peephole connection in the cell [45]. It is a critical problem in the vanilla RNN structure as is described in [50]. With the application of convolutional LSTM, the sequence length of the FEA computational results handled to the neural network model is variable. This makes the tracking of long-term data dependencies possible to realize. It offers the network to share parameters across the FEA result sequence.

### 3.3. Training Data

#### 3.3.1. Post-Processing Images of FEA Computation

**Figure 3:**
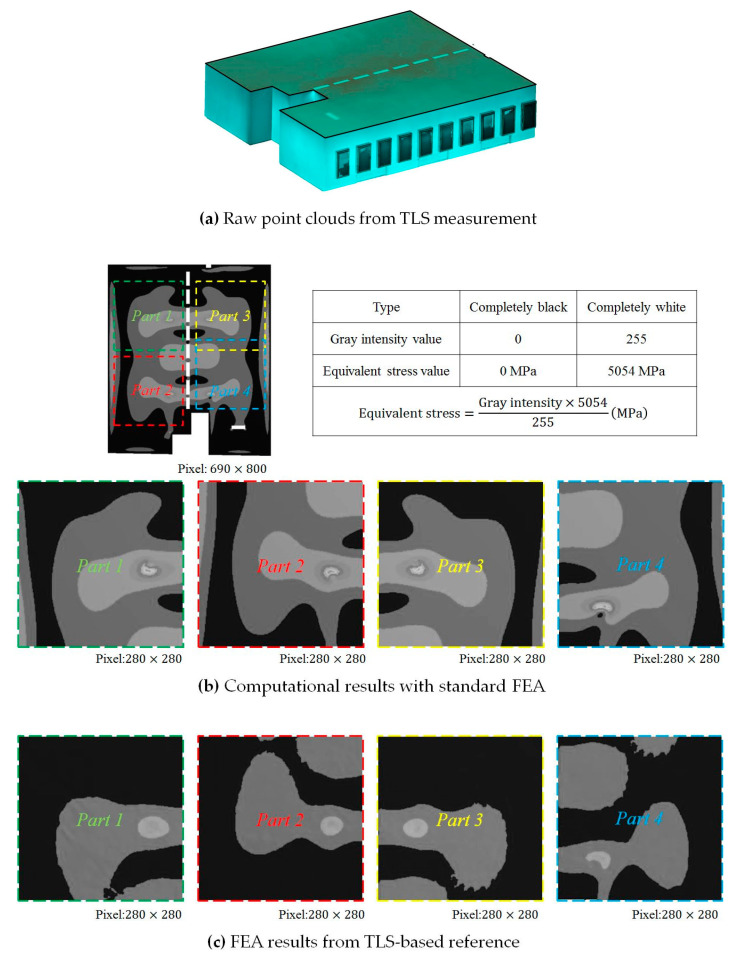
Images of point clouds and finite element analysis (FEA) equivalent stress results.

The basis of the calibration of the standard FEA computation is the simplified model which ignores all irregular deformable details. Simple flat planes generate the simplified FEA model. The positive gain of the application regarding the standard FEA computation is the efficiency of the computation. The next task is to improve the accuracy of the efficient computation in this research. Hence, the reference result is based on a parametric FEA model is applied. The accurate parametric geometry is reconstructed with the combination of TLS point clouds data, as is shown in Figure 3a. The details regarding point clouds can be found in the previous work [69]. The accuracy of the parametric model depends on two parts which are the accurate geometry scanning with TLS [79] and the accurate description with B-spline surface [80]. B-spline approximation is beneficial in fitting scattered points with the advantage of surface continuity representation [80]. Accordingly, a selection is made of the parametric FEA computation as the monitoring TLS-based reference method regarding the model accuracy in this research. Details regarding the parametric FEA computation can be found in [69]. The FEA computation is conducted under continuous static loadings which are surface-based distributed on the roof of this building. The researched object in this research is the roof surface in the model of [69]. The static loading begins from 16 kN. Each loading increases linearly by 0.2 kN. The total number of loadings is 141. The equivalent stress value forms the basis for the value analyzed. All geometric details, parameters, and boundary conditions are related to Section 3.1 of [69]. The object being calibrated is the roof part.

The equivalent von-Mises stress value is a positive value output directly from the FEA computation. Utilizing original equivalent stress information to describe the FEA post-processing results can produce a similar color map contain an alternate range due to the various interims of deformation results under various loadings. This will introduce various gray hues corresponding to the same equivalent stress values in various image descriptions. This will affect the training effect and efficiency if the color intensity maps of these images are not unified. Accordingly, the process of the FEA computation images unifies the gray intensity epochs with the same color map distribution according to the equivalent stress range in this example, shown in Figure 3b,c. Alternatively, Figure 3b shows the computational results from standard FEA computation, while Figure 3c presents the FEA computational results from TLS-based reference in Figure 3a. The length of the training sequence is 16. It means the utilization of the previous 8 epochs predicts the next 8 adjacent epochs in the future. The computation results contain 141 groups of epochs. The gray intensity value 0 is corresponding to the zero equivalent stress value and the gray intensity value 255 is corresponding to the largest equivalent stress value. Hence, the completely black color indicates the smallest equivalent stress, while the completely white color indicates the largest equivalent stress in this research, as is shown in the relative value comparison table of Figure 3b. The complete white is invisible and cannot be shown in the figure with the completely white content background even through it is easy to be implemented in the procedure. As a result, the comparison table of Figure 3b provides the relative value comparison between the gray intensity and equivalent stress value.

All equivalent stress values in all epochs shown in this research can be converted and obtained by the proportional relationship between the gray intensity and equivalent stress in the table. There are 690×800 pixels in each original image. Four regions where the large equivalent stress develops from can be found. Therefore, the monitored surface is divided into four separated parts for the NNs computed later. It is obvious to find the blank rectangular regions in the figure with 690×800 pixels. As a result, the final refined images have 280×280 pixels to avoid the blank regions and retain the main variable features of the monitored surface. Meanwhile, the refined regions contain the most obvious variable features.

Figure 3b,c indicates a comparison example of four different parts corresponding to the same epoch. The same epoch represents the same static loading and equivalent stress response in the FEA computation. Results indicate apparent deviation between the standard FEA computation and the TLS-based reference. As a result, it is significant to calibrate the efficient but inaccurate standard FEA computation. The calibration of the standard FEA can be implemented in either stress, strain, or deformation results or all of them. The equivalent stress calibration tests and applies the implementation of the methodology in this research.

#### 3.3.2. Data Processing

**Figure 4:**
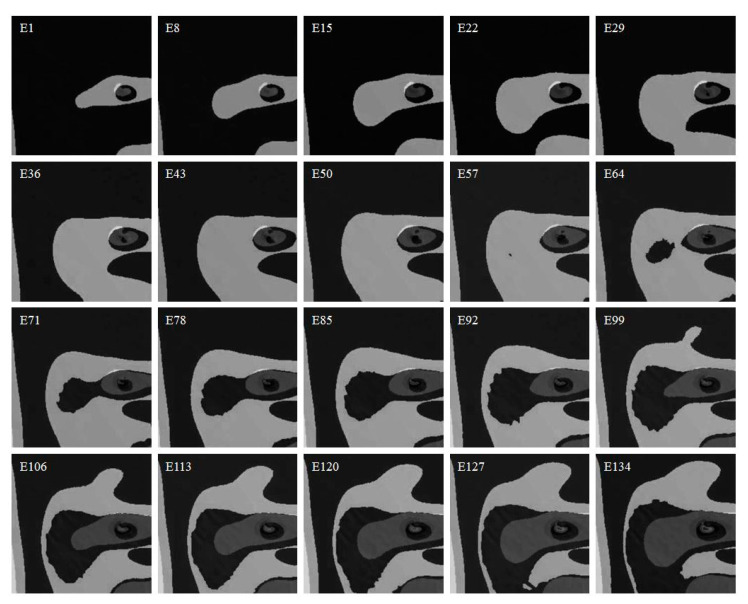
Equivalent stress deviation images of Part 1 in gray.

Figure 4 presents an overview of the development of equivalent stress deviation epoch examples in Part 1 regarding the monitored surface. E in the left-upper side of images stands for the epoch. Twenty epoch samples are presented in Figure 4. The shades of different gray colors illustrate the equivalent stress characteristics of different models. The equivalent stress value can be intuitionally indicated by the gray scale map with shades of gray. The different equivalent stress values vary a lot in different parts of the monitored images. The equivalent stress values of different images appear more similar when the color part is closer to the black color or value 0. As is shown in Figure 4, there are a number of similarities among the equivalent stress distribution when the static loading is low. With the development of the static loading, the FEA computation of equivalent stress results indicate dramatic deviations. Hence, the equivalent stress deviation increases gradually.

The equivalent stress of the monitored surface is computed in this research. Section 4.1 shows the comparison and analysis of the deviation in different parts. The extracted deviation images regarding the object equivalent stress are represented as three-dimensional dataset with width, height, and channel. The width and height have 280 pixels in this model. And the channel is 1. There are four parts computed in the model, as is shown in Figure 3. The deviations dataset in each part contains 140 sequences. All sequences are divided in 60% training sequences, 20% testing sequences, and 20% validation sequences, which refers to the grouping method in [45]. All sequences are 16 deviation epochs long, including 8 epochs for the input and 8 epochs for the prediction.

## 4. Results and Discussion

This section mainly presents the important results. Some discussions and analysis are conducted in details. It begins with the presentation of MSE results and the analysis based on MSE regarding the original FEA computational results. Some examples show a visual representation of the prediction results. The MSE and structural similarity (SSIM) of the predicted results are calculated. We reveal the histogram and frequency results of image intensity in details. The evaluation of the predictions from the deep learning model are carried out based on the calculations of above results. Meanwhile, the FEA calibration is optimized by the threshold control. Finally, the analysis regarding the normal FEA calibration and optimized calibration is carried out.

### 4.1. Analysis of FEA Results

(24) $$MSE=\frac{1}{mn}\overset{m-1}{\underset{i=0}{\sum }}\overset{n-1}{\underset{j=0}{\sum }}{\left[a\left(i,j\right)-b\left(i,j\right)\right]}^{2}$$

Within this section, the MSE value is used as a quality estimator of deviation values [81] and can be applied to evaluate the deviation between two FEA images. Equation (24) describes the formulation method. a(i,j) and b(i,j) are the gray intensity values to their corresponding equivalent stress values$\;a_{t}$ and$\;b_{t}$ in Figure 1. MSE is the most widely applied metric by averaging the squared intensity deviations of distorted and TLS-based reference image pixels, along with the related quantity of the peak signal-to-noise ratio [82]. In the current research, MSE values of different parts are calculated to compare the deviation between standard FEA and reference results.

**Figure 5:**
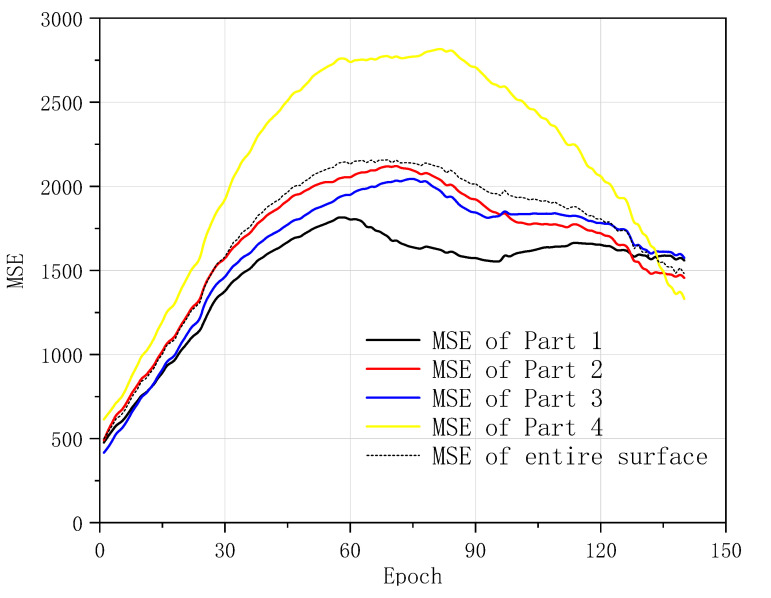
Mean square error (MSE) values between standard FEA and the terrestrial laser scanning (TLS)-based reference result.

Different parts of the monitored surface equivalent stress reveal the MSE values, shown in Figure 5. There are a number of important deviations between the standard FEA results and TLS-based reference results. The MSE value of the epoch increases with the development of the static loadings. It trends to drop down in the middle of the horizontal axis. The beginning positions of the decline trend vary from each other in different parts. The MSE value of Part 1 performs best within the four parts, which means the deviation of Part 1 between the standard FEA result and reference result is minimal. The MSE values of Parts 1, 2, and 3 are smaller than the MSE of the entire monitored surface. The MSE of Part 4 performs extremely abnormal in the comparison to the others. It indicates that the standard FEA computation with simplified geometry demonstrates the lack of mechanical behavior in the simulation process regarding Part 4 while it is compared with the TLS-based FEA computation. This confirms the inadequacy of the simplified model. Meanwhile, it verifies the necessity of the calibration and optimization regarding the simplified or standard FEA computation in the mechanical application.

The fluctuation of MSE curves in Parts 1, 2, and 3 performs gentle within the range of epochs 90 to 130. It reveals the equivalent stress performance is very similar during these static loadings in both standard FEA computation and TLS-based reference results. It is confirmed that the mechanical development of the object can be fitted by the standard FEA computation in some stages and monitored regions correctly and efficiently.

### 4.2. Evaluation of Prediction

**Figure 6:**
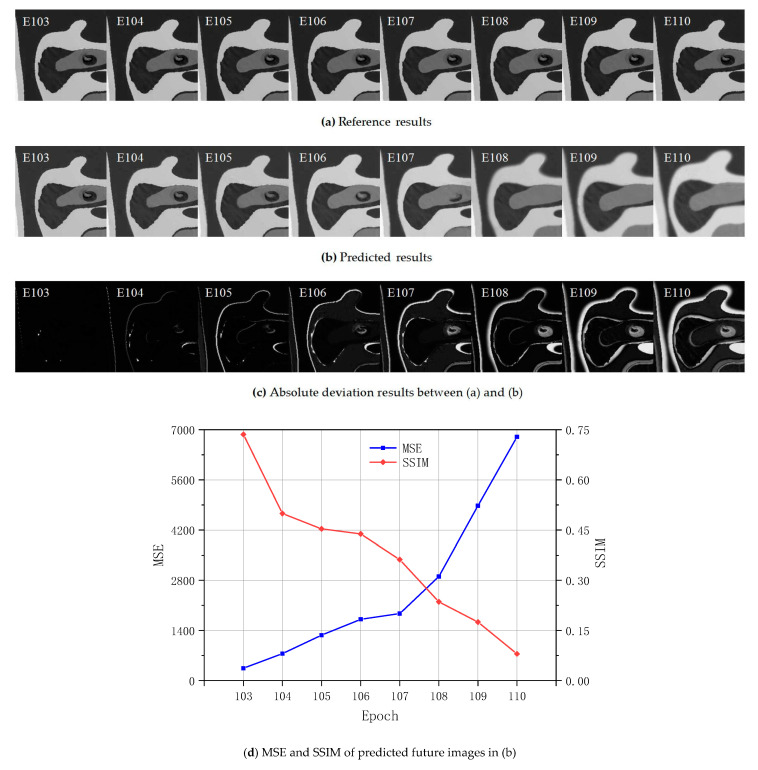
Comparison regarding the reference and predicted results.

Figure 6 shows the comparison between the TLS-based reference deviation images and the predicted results. The current inputs from epochs 95 to 102 provide the basis for the outputs of the predictions from epochs 103 to 110. This indicates that we apply eight input epochs to predict eight future epochs. This research tests five strategies regarding the number of the input and output epochs, in which the numbers of input and output epochs are equal, including 4, 6, 8, 10, and 12. The number of 8 is finally applied according to the best model training performance in this condition, in which the MSE is smallest. The overall development trend of both results in Figure 6a,b is similar, which proves that the deep learning results are close to those in the FEA prediction process. The overall feature of the deviation images regarding the general contours are followed well by the predicted ones, especially in epochs 103, 104, 105, and 106. In terms of contour details, the first two predictions, where bump features follow the initial data well, perform better. This can bring excellent equivalent stress monitoring on the surfaces in the static loading experiment. However, the predicted deviation images trend to miss some detailed features in the learning process. There are some deviations in epochs 107, 108, 109, and 110. The absolute deviations in Figure 6c prove this.

The absolute deviations between the predicted and reference results become increasingly obvious with the development of the epochs. In contrast to earlier predictions, apparent evidence of contour blur is detected. The later prediction process ignores all contour boundaries. Small features which vary slightly and slowly in earlier future predictions trend to vary fast and disappear. The prediction of FEA equivalent stress deviation images confirms and verifies the future prediction feasibility of the application of the methodology proposed. Consequently, the requirement of the prediction steps can be determined to take the earlier four predictions in the engineering application while applying the methodology in the static loading.

Figure 6d shows the calculation of the MSE and SSIM. SSIM is a method for predicting the perceived quality of digital pictures to measure and evaluate the similarity between two images [83]. Equation (25) shows the formulation of SSIM regarding two epochs a and b [83]. More details of SSIM can be found in [82,83]. $\mu_{a}$ and $\mu_{b}$ are the local means. $\sigma_{a}$ and $\sigma_{b}$ are the standard deviations.$\;\sigma_{ab}$ is the covariance of a and b.$\;C_{1}$ and$\;C_{2}$ are two variables. $C_{1}={\left(k_{1}L\right)}^{2}.\;C_{2}={\left(k_{2}L\right)}^{2}.$ Here, $k_{1}=0.01.\;k_{2}=0.03.$ And L=255.

(25) $$SSIM\left(a,b\right)=\frac{\left(2\mu_{a}\mu_{b}+C_{1}\right)\left(2\sigma_{ab}+C_{2}\right)}{\left(\mu_{a}^{2}+\mu_{b}^{2}+C_{1}\right)\left(\sigma_{a}^{2}+\sigma_{b}^{2}+C_{2}\right)}$$

The blue line in Figure 6d presents the trend of the MSE. The red line presents the trend of the SSIM. The process occurs between the original deviation results and predicted results for the calculation of both values. Figure 6d shows that the MSE of the first future prediction is 346. MSE values improve with the development of the processed epochs. The MSE values vary slightly in epochs 103, 104, 105, 106, and 107. MSE varies significantly after prediction of epoch 107, which reveals that the prediction regarding future equivalent stress images after epoch 107 should be considered as significantly too large in the static loading experiment. Deviations after epoch 107 increase greatly. This indicates that the further the predicted images are in the future, the higher the deviations should be. The application of the SSIM index improves the evaluation of traditional methods. SSIM is a decimal value between −1 and 1. The value of 1 proves perfect structural similarity within two images. The first future prediction contains a SSIM of 0.74. It indicates strongly inter-dependent pixels of the predicted image. With the development of the epochs, the SSIM reduces, especially after epoch 106. Regarding the predictions, Epochs 103 to 106 are more accurate. Accordingly, we discuss the first four future images in the predicted results more in the following.

After comparing the results from some published references [45,84], we find that the blur problem is obviously general with the application of LSTM when the predicted future sequence is too long. This is also proved in [51]. Moreover, a very long predicted sequence in static loading experiment is undesirable. Other sequences regarding the prediction parts perform the similar variable law as in Figure 6d. The further the predicted results are in the future, the higher the deviation from deep learning trends to be. Consequently, the variable law in Figure 6d is the feature within a large sequence range in all sequences, which is also proved in all predicted examples with the LSTM method in [51]. Figure 9 in Section 4.3 can confirm it.

**Figure 7:**
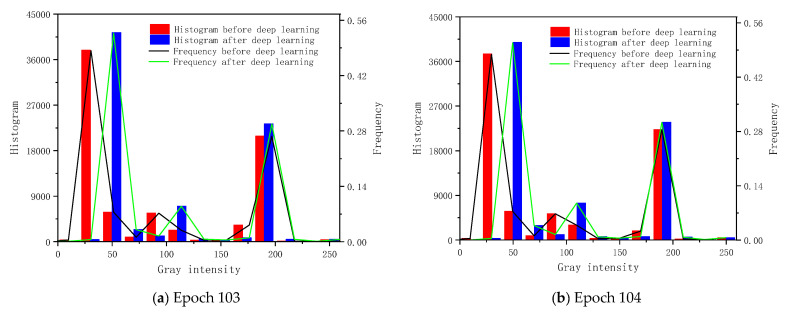

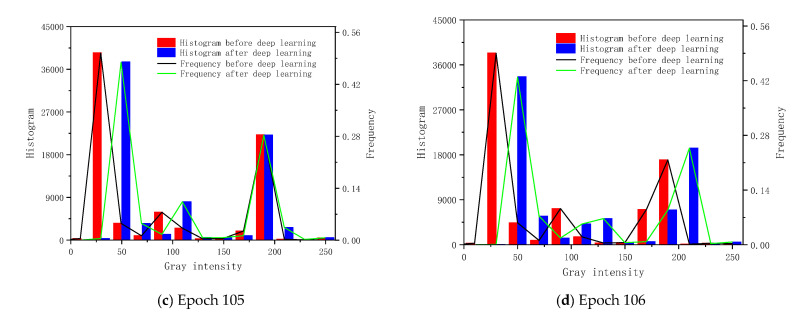
Histogram and frequency of image intensity.

With the combination of Figure 6a,b, the median value in Figure 7 reveals the blur problems in the long sequence study period. The intensity distribution of the equivalent stress deviation images regarding the first four epochs in Figure 6a,b focuses on the low and high intensity part. The frequency in the median value interval is low. Accordingly, the predicted images in these four epochs perform well in the boundary and different gray region description of the contours. Meanwhile, this also explains why the TLS-based FEA calibration is focused on the next four steps in static loadings in Section 4.3.

### 4.3. Discussion of Calibration

**Figure 8:**
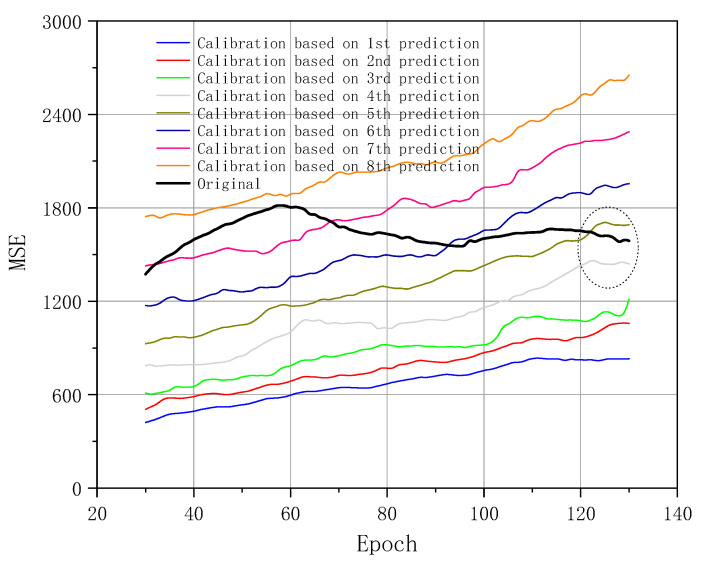
MSE results regarding FEA computation after calibration.

Figure 8 shows MSE values of the FEA future outputs after the calibration. The original curve is the comparison curve which is corresponding to the results of Part 1 in Figure 5 from epochs 30 to 130. When the MSE value is reduced, the calibrated result is improved. The MSE curves apparently show that the calibration based on the first four prediction results of deep NNs reduces the MSE values of the original FEA computations. The MSE increases with the development of the deep calibration in long sequences, and the MSE rises up. The reason is that the equivalent stress image contains larger variable regions with the development of the static loadings. The MSE values are pretty close to the original curve data after epoch 120 which is highlighted as a dotted circle. As discussed in Section 4.2, the further the predicted results are in the next steps, the higher the deviation from deep learning trends is. The MSE values in the calibration relate to those in the prediction part. The MSE values in the calibration after the fourth prediction are higher than the first four results. The calibration accuracy is increasingly worse after the calibration on the basis of the deeper future outputs after the fourth prediction. Accordingly, we do not discuss the calibrated results after the fourth prediction in this section anymore. There is an obvious improvement of the accuracy after the intelligent calibration of FEA computations. Meanwhile, the most obvious calibration results relate to the first prediction of the NNs model.

**Figure 9:**
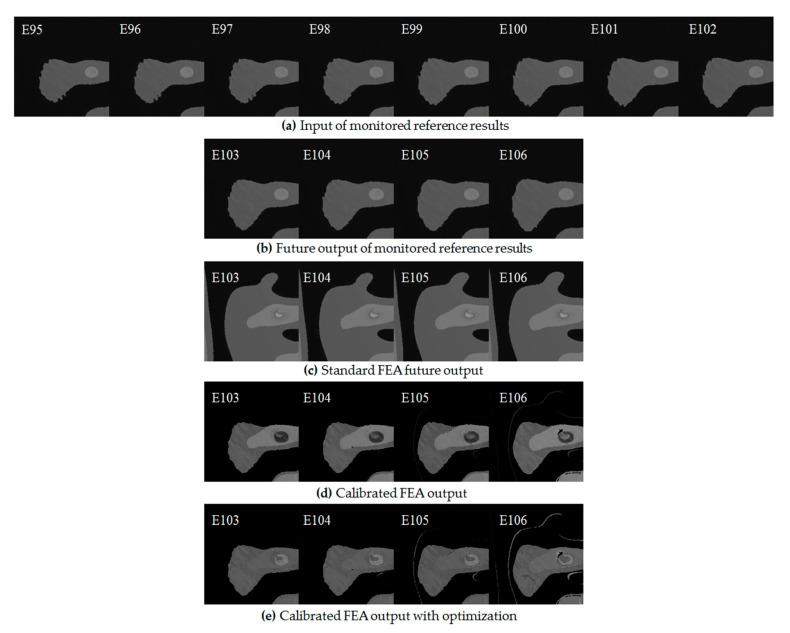
Calibrated FEA results.

Both the deviation image learning and the simplified FEA computation results provide the basis for the calibration of the FEA computation, as the methodology section and Figure 1 describe. This manuscript takes from the epochs 95 to 102 as the input of the TLS-based reference results in this figure sample, as is shown in Figure 9a. Hence, according to the explanation in Section 4.2, the next four images, which are epochs 103, 104, 105, and 106, are the future output from the monitored results, as is shown in Figure 9b. These are also the ground truth in this research. Figure 9c shows the original future output from the standard FEA computation, which is also the object required to be calibrated in this figure. Figure 9d shows the calibrated results applying the proposed methodology.

Figure 9d shows that the calibrated future FEA results are improved significantly with the comparison of the original images in Figure 9c. The boundary follows with the TLS-based reference feature correctly, especially in the first three calibrations. Moreover, the calibrated output results describe the deformable surface details well. There are more equivalent stress changes around the deformable surface according to the intensity of the equivalent stress image. The deformation region with higher equivalent stress detection could show crack behaviors when the loading force is very high in the engineering application. However, the original FEA computation before calibration cannot detect the deformable surfaces. Accordingly, the future output after calibration with deep NNs can perform effective roles in health monitoring in the engineering application.

The obvious difference between Figure 9b,e is that Figure 9e contains a horizontal highlighted bar region in the gray part. It makes the calibrated results inaccurate in some parts of the monitored surface. Accordingly, the calibration is also optimized by threshold controls, as is shown in Figure 9e. The solution is to find out the deviation between the stronger and normal intensity. The edge detection function in Python detects the boundaries between different regions. Since the gray intensity is different in each pixel, one can calculate the average intensity values of the stronger and normal intensity region. The deviation between the average intensity values in both regions generates the threshold control. Subtracting the threshold control value weakens the gray values within the region with stronger intensity and a close intensity corresponding to the TLS-based reference value. Meanwhile, the threshold control strategy in the middle circle region also strengthens the weak intensity. The same method as the previous one calculates the threshold control value. It is obvious to find that the optimized calibration in Figure 9e performs better than the normal calibration in Figure 9d.

The main error of the calibrated output is mainly focused on the gray intensity compared with the TLS-based reference output, especially in the middle region with a horizontal bar shape regarding the equivalent stress images. The deviation of the gray intensity after the deep learning causes this. The possible solution is to separate the gray region in an independent deep learning. One utilizes the separated region after calibration to replace the inaccurate region to generate a new calibrated result.

The object in the entire research of the calibration is taken to be the contour with boundary, as is shown in Figure 9. This indicates that the gray contour has been divided into different intensity range with gray bands. The disadvantage of this method is the discontinuity of the equivalent stress description. During this research, the smooth gray contour is also investigated as a comparison. However, one cannot predict or calibrate the variable features without the obvious gray band correctly in the convolutional LSTM model. As the contour boundaries are added, the deep NNs are able to learn the variable characteristics in equivalent stress images. One possible method to perform more continuous contour description is to divide the contour into more gray bands if necessary. Another method is to carry out an interpolation calculation in different gray intensity boundary regions.

**Figure 10:**
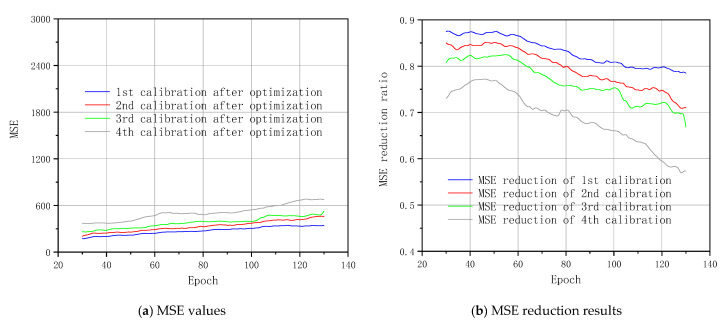
MSE curves regarding FEA calibration after optimization.

Figure 10 reveals the MSE values of FEA future output after optimized calibration. It proves that the threshold control method to decrease the MSE values is effective. Meanwhile, it indicates that the deviation in the middle horizontal bar region is the main source of the MSE in Figure 9. The optimization is based on the threshold control method which is described in the explanation of Figure 9. Figure 10 focuses on the first four calibrations. Calibration values based on the first three prediction results are relatively close and kept within a low MSE level after the optimization. Based on the examples in Figure 9, the MSE reduction ratios in the first three calibrations are below 66%. The original curve data of Figure 8 is the basis for the calculation of the MSE reduction ratio here. The threshold control apparently decreases the MSE values of the fourth calibration significantly, which is close to the original curve in the circle mark of Figure 8. The MSE reduction ratio in this part is also below 57%. An overall improvement of the calibration quality occurs. With the combination of Figure 9d,e, the large region in the middle horizontal bar of the image provides the main basis for the improvement. Calibration and optimization of the region range and equivalent stress value are calibrated and optimized significantly. Accordingly, the optimized calibration of the FEA future output benefits from the threshold control.

## 5. Summary and Conclusions

The current research aims to explore effective methods and improve the depth in calibrating the continuous static FEA computations. Machine learning proves to be the successful method. However, the sequence-based FEA calibration is still unsolved. Accordingly, we selected the convolutional LSTM model to calibrate the future sequence results corresponding to adjacent continuous loadings in the FEA. We addressed the intelligent FEA calibration problem in two ways: (1) by using the normal FEA computation to generate the original future output, and (2) by using the estimation and prediction of deviation results between the standard FEA results and the TLS-based reference. Training of the previous deviation results and predicting the future deviations results took place. By integrating the results from the simplified FEA and predictions from the convolutional LSTM model, the future FEA results are generated as the sequence-based representation. The threshold control finally optimizes the calibration. The paper is composed of five main sections. Section 1 introduces the development background of FEA and deep learning. Novel combination approaches and the future sequence prediction techniques are described. Section 2 discusses the motivation of this manuscript, which leads to the main issues to be discussed. It also describes the general framework regarding how to realize the proposed methodology. The LSTM model is introduced and discussed in details. Section 3 presents the mathematical description of the model and methodology, the main implementation of LSTM, and the training of the intelligent FEA model. Section 4 discusses and analyzes the results regarding the final calibration. Finally, Section 5 relates the summary and draws main conclusions of this research. Based on the results, the main points of this research are concluded as the following:

1. Compared with the TLS-based reference, the standard FEA computation with a simplified geometry reveals large MSE values regarding the equivalent stress all through the computation process. There is a reduction of the final MSE results when applying the convolutional LSTM model and TLS-based reference. It ensures the improvement of the accuracy in the proposed methodology.

2. The quality of predictions reduces with the increase of the static loadings. When the size of prediction step is too long, it reveals the blur problem in predicted frames. The blur problem is focused on two aspects. One is based on the contour boundary. Another is based on the blur of the entire predicted images in the later long-term or long-step prediction. Additionally, the unexpected improvement of the image color intensity in predicted results indicates the disadvantage.

3. Calibrating the FEA future sequences reduces the final MSE value significantly. The first four future predictions are very accurate. We suggest taking the first four predictions as the calibration sequence in the calibration part. The calibration output performs excellently corresponding to the TLS-based reference results. The contour boundary in the calibration is described very close to the true values. The threshold control provides an effective optimization to the calibration results. The MSE value is further reduced by the threshold control. It ensures the effectiveness of the proposed methodology.

4. The depth issue of the FEA future calibration is addressed successfully through the sequence-based LSTM. However, the amount of training dataset needs to be increased further. This can further enhance the reliability of the proposed methodology.

Additionally, the current FEA calibration method is different from the popular CNN-based FEA computation. It reveals more possibilities and potentials of deep learning methods to be applied in the FEA calibration. In the future, we will focus on the sequence-based FEA calibration while exploring more methods to improve the prediction depth and the calibration accuracy in the engineering application regarding continuous static loadings.

## Abbreviations

The following abbreviations are used in this manuscript:

FEA	Finite element analysis
LSTM	Long short-term memory
NN	Neural network
CNN	Convolutional neural network
RNN	Recurrent neural network
TLS	Terrestrial laser scanning
MSE	Mean square error
SSIM	Structural similarity

## Nomenclatures

Nomenclatures of different parameters used in equations of this manuscript are defined as following:

$a_{t}$	Output result from standard FEA computation before calibration
$b_{t}$	Output result from reference method before calibration
$x_{t}$	Deviation between standard FEA and reference outputs before calibration
${\hat{x}}_{t}$	Predicted deviation value with deep learning
${\hat{b}}_{t}$	Calibrated output results
σ	Sigmoid function
W	Weight of the respective gate neurons
b	Bias of the respective layer
$f_{t}$	Output from the forget gate layer of LSTM
$i_{t}$	Output from the store gate layer of LSTM
$c_{t}$	Output from the update gate layer of LSTM
${ο}_{t}$	Output from the output gate layer of LSTM
$y_{t}$	Predicted output from LSTM model
$h_{t}$	Hidden state in LSTM model
${ℱ}_{t}$	Output from the forget gate layer of convolutional LSTM
${ℐ}_{t}$	Output from the store gate layer of convolutional LSTM
$C_{t}$	Output from the update gate layer of convolutional LSTM
$O_{t}$	Output from the output gate layer of convolutional LSTM
${ℋ}_{t}$	Hidden state in convolutional LSTM model
μ	Local mean in SSIM
C	Variable in SSIM
$\sigma_{ab}$	Covariance of a and b
